# Resource Allocation to Massive Internet of Things in LoRaWANs

**DOI:** 10.3390/s20092645

**Published:** 2020-05-06

**Authors:** Arshad Farhad, Dae-Ho Kim, Jae-Young Pyun

**Affiliations:** Department of Information and Communication Engineering, Chosun University, Gwangju 61452, Korea; arshad@chosun.kr (A.F.); wireless@chosun.kr (D.-H.K.)

**Keywords:** LoRaWAN, spreading factor assignment, inter-SF and intra-SF interference, urban environment

## Abstract

A long-range wide area network (LoRaWAN) adapts the ALOHA network concept for channel access, resulting in packet collisions caused by intra- and inter-spreading factor (SF) interference. This leads to a high packet loss ratio. In LoRaWAN, each end device (ED) increments the SF after every two consecutive failed retransmissions, thus forcing the EDs to use a high SF. When numerous EDs switch to the highest SF, the network loses its advantage of orthogonality. Thus, the collision probability of the ED packets increases drastically. In this study, we propose two SF allocation schemes to enhance the packet success ratio by lowering the impact of interference. The first scheme, called the channel-adaptive SF recovery algorithm, increments or decrements the SF based on the retransmission of the ED packets, indicating the channel status in the network. The second approach allocates SF to EDs based on ED sensitivity during the initial deployment. These schemes are validated through extensive simulations by considering the channel interference in both confirmed and unconfirmed modes of LoRaWAN. Through simulation results, we show that the SFs have been adaptively applied to each ED, and the proposed schemes enhance the packet success delivery ratio as compared to the typical SF allocation schemes.

## 1. Introduction

A long-range wide area network (LoRaWAN) is a wireless technology designed for low-power wide area networks [1]. Owing to its long range, low power, low cost, and open business model, it is widely adopted as one of the alternatives for the internet of things (IoT). The IoT comprises an enormous number of end devices (EDs) scattered over a wide geographical area, thus creating a high-density and large-scale environment. Recently, both academia and industry have been attracted to LoRaWANs because of their recent developments and applicability in the IoT environment. An LoRaWAN defines the media access control (MAC) protocol and architecture for layer two communication. At the same time, LoRa specifies the physical layer using chirp spread spectrum (CSS) modulation for long-range and low-energy communication. 

In a massive IoT network, EDs serve various applications subject to different constraints, i.e., length of a packet, network delay, data rate (DR), acknowledgment delay, reliability, throughput, etc. To increase the spatial diversity of the applications, LoRaWAN uses three types of ED classes: *A*, *B*, and *C*. The class *A* EDs have very high energy efficiency and provide bi-directional communication using ALOHA; they, however, support only unicast communication. In class *A*, an ED opens a receive window (RW) for the downlink (DL) to receive an acknowledgment (ACK) confirmation from a network server (NS) after a confirmed uplink (UL) transmission. Class *B* EDs are battery powered and provide bi-directional communication. These devices support unicast and multicast transmissions; furthermore, they have more RWs than class *A* EDs. The class *B* EDs are synchronized by a beacon frame transmitted from the GW. On the other hand, class *C* EDs use more power than class *A* and *B* as they listen to the channel all the time. 

To improve the spectral efficiency and enhance the capacity of the network, LoRaWAN uses six spreading factors (SFs) with CSS modulation. The allocation of SFs to the EDs affects the transmission success, i.e., a higher SF allocation induces a long-distance coverage. At the same time, it indicates a lower DR and higher time-on-air (ToA). These EDs randomly select a channel for UL transmissions using ALOHA. Owing to the use of ALOHA, the LoRaWAN suffers from packet collision caused by the same SF over the same channel (intra-SF interference) or different SF over the same channel (inter-SF interference), thus resulting in a high packet loss. In a confirmed mode, this packet loss forces ED retransmissions, where each ED increments its SF each time two successive retransmissions fail [1,2]. It is assumed that the retransmission failure occurs owing to poor connections; hence, a higher SF is expected to increase the packet success ratio, because it enhances the receiver sensitivity [3]. However, after a high SF is assigned, the ToA of the ED packet increases, and the collision probability of ED packets increases drastically. Because many EDs switch to higher SFs, it causes an avalanche effect [4]. To overcome this effect in the network, we propose a scheme called the channel-adaptive SF recovery that increments or decrements the SF based on the retransmission of the ED packets in the network.

Moreover, to overcome the impact of intra- or inter-SF interference in the LoRaWAN network, some of the approaches allocate SF according to the distances from the GW in unconfirmed mode [4,5,6,7]. These SF allocation schemes are mainly based on the fixed-width SF rings (also known as ring-based approaches) such as equal distance-based [4], GW sensitivity-based [5,6], and signal-to-noise ratio (SNR)-based [7]. Such ring-based approaches can perform better in an unconfirmed mode, where no DL ACK message is required from the GW. On the other hand, in the confirmed mode, each packet is acknowledged with a DL message. Hence, in an environment of heavy traffic load, the network becomes more congested owing to the bi-directional communications and retransmissions, leading to significant packet loss. Thus, the ring-based SF allocation approaches exhibit a less efficient SF allocation, resulting in a lower packet success ratio in situations of heavy bi-directional traffic load. To improve the packet success ratio, we propose to allocate SFs to EDs based on the ED sensitivity by measuring the received power that a GW would receive from the ED. The proposed SF allocation is different from the ring-based approaches, where the proposed scheme assigns different SFs to EDs having the same distance from the GW.

The contributions of this paper are threefold: (1) This paper provides an in-depth survey and analysis of inter-SF and intra-SF interferences. Through simulation results, we show the collision overlap time occurred due to the collision of packets with intra- or/and inter-SF interferences. Additionally, we show the impact of both intra- or inter-SF interferences on average end-to-end and ACK delays using our proposed schemes under path loss, shadowing, and building penetration losses in the LoRaWAN network. (2) When a packet loss occurs due to interference, each ED steps up its SF, resulting in a higher SF. It is shown that higher SF is highly vulnerable to interference, thus the proposed scheme based on the channel status decrements and increments SF to lower the chances of collisions. (3) Unlike fixed-width SF rings-based approaches, our proposed ED sensitivity-based scheme assigns different SFs in the same region based on the signal strength received at the GW. This behavior makes it different from the existing ring-based approaches and helps to lower the impact of interference.

The rest of this paper is organized as follows: Section 2 surveys the recent research work in the area of resource allocation. The LoRaWAN network model, comprising link measurement and link performance, is presented in Section 3. Section 4 presents the proposed SF allocation schemes. The experimental results and analysis of the proposed SF allocation schemes are presented in Section 5, while Section 6 concludes this paper.

## 2. Literature Review

In this section, we briefly survey the existing resource allocation solutions in the LoRaWAN network. We divide these solutions into three broad categories: Interference-based, link- and system-based, and mathematical model-based approaches. The primary aim of these approaches is to enhance the packet success ratio.

### 2.1. Interference-Based Approaches

For the experiments on interference and the capture effect in a LoRa channel, an error model is considered. It can be applied to experiments monitoring the interference between communications using different SFs, as presented in [8]. This work shows three different approaches for allocating an effective SF: (i) A random SF assignment approach, which allocates the SFs based on a uniform distribution; (ii) a fixed SF assignment approach, which allocates static SFs during the initial deployment; and (iii) the packet error rate (PER) approach, which assigns the lowest possible SF using an error model based on additive white Gaussian noise (AWGN). The PER mechanism achieves a higher transmission success ratio by finding an appropriate SF than both random and fixed SF-based allocation approaches. However, the authors in [8] did not take into account the inter-SF interference, shadowing, and building penetration losses. Another work based on collision types to manage the SF is presented in [4]. There are two types of collisions observed during communications: (a) Collision of two packets with the same SFs, and (b) collision of two packets with different SFs. The primary aim of this collision-aware SF assignment method is to improve the PER by enhancing channel fairness. Firstly, this method categorizes the EDs to form groups based on the radio frequency (RF) coverage and path loss, wherein each group uses a distinct channel. Secondly, the lowest SF is selected and allocated to each group based on the observed cumulative interference ratio (CIR). The proposed scheme in [4] decreases the PER up to 42% overall. However, this improved performance comes at the cost of increased energy consumption compared to the conventional distance-based scheme [5], and the simulation analysis is only limited to the collision case (a). An SF assignment mechanism for the LoRaWAN network introduced in [9] aims to improve the success ratio by reducing the interference caused by the same SFs and channels. To categorize the interference of two packets, authors in [9] measures the signal-to-interference-plus-noise ratio (SINR) of packets transmitted with the same SFs over the same channels. In the simulation, if the measured SINR is larger than the threshold limit, the packets survive the interference. Otherwise, the packets are lost to the interference. However, authors in [9] does not take into account the interferences between packets with different SFs over the same channel, as these SFs are not entirely orthogonal [10,11,12,13,14]. A similar scenario related to the impact of non-orthogonality concerning the data extraction rate is presented in [11]. The study in [11] reveals that the impact of non-orthogonality is limited when the traffic load in the network is low. The authors of [11] also show that higher SFs (e.g., 10, 11, 12) are more venerable to interference due to their high ToA. It is also assumed in [15] that a collision occurs due to the simultaneous transmission of the packets over the same SF and channel. However, the analysis in [15] is limited to the case with an SF of 12 only. Furthermore, the effect of the non-orthogonality concerning SFs is further deliberated in [16], where it computes the probabilities of successful UL packet and coverage based on [11]. The study in [16] shows that UL packets transmitted with different SFs can collide as long as their received power at GW is different. It also reveals that the impact of inter-SF interference can be high enough in a significantly large network. However, in a short distance (i.e., less than 1 km), the effect of inter-SF interference is less. Additionally, the scalability of LoRaWAN under the impact of interference in the absence of DL traffic was studied in [17]. The testbed and simulation results concluded that one of the two packets can be received if the headers of both packets do not collide. In contrast to previous works, authors in [18] considers both inter- and intra-SF interferences. The authors in [18] aimed to maximize the LoRaWAN network capacity by optimizing number of EDs for a given SF. The numerical results showed that the proposed method in [18] maximizes the LoRaWAN network capacity up to 700%, as compared to the equal number of EDs per SF strategies.

### 2.2. Link- and System-Based Approaches

The capacity of an LoRaWAN in terms of the maximum number of EDs that can communicate in a single and multi-cell scenario is analyzed in [3] under realistic traffic conditions in the simulation and the testbed. Furthermore, use cases for LoRaWAN are shown based on the traffic type, payload size, and minimum required packet success ratio (SR) [19]. It is certain that increasing the network capacity in terms of the offered traffic in all the use cases can provide a satisfactory SR. Furthermore, [3] shows that the SR increases with the number of GWs. In the unconfirmed mode, the traffic load has a low effect on the SR, as the geometric coverage plays a significant part, resulting in an SR of 55% or 93% depending on the number of GWs. Furthermore, in [3], the intra-SF interference is limited to SF values of 7, 9, and 12. To improve SR, each ED is allocated with an SF based on the GW sensitivity by measuring the received power that a GW would receive from the ED during the initial deployment, as presented in [5]. Based on the measured received power, each ED picks an SF based on the GW sensitivity threshold defined for every SF. However, this work only focuses on enhancing the packet success ratio in the UL transmission and does not take into consideration the DL transmission. 

The reliability and scalability of the LoRa (RS-LoRa) scheme aims to enhance the reliability and scalability of an LoRaWAN under an ideal network situation [20]. RS-LoRa works in two phases: In the first phase, GW groups the EDs within its coverage by obtaining the received signal strength indicator (RSSI) and SF on each channel. In the second phase, EDs get their SFs, transmit power, and a channel based on the RSSI. This grouping based on the RSSI decreases collision by choosing a suitable SF, and enhancing the network reliability, scalability, and capture effect. Another work presents a system-level simulation with heterogeneous traffic in [21]. The primary purpose of the work in [21] is to enhance the SR by allocating possibly the lowest SFs based on the SNR of the ED packet. Hence, the scheme in [21] decreases the ToA for each ED and reduces the probabilities of collision by suitable SF allocation. A similar approach called an adaptive spreading factor selection (ASFS) algorithm aims to allocate SFs to EDs in the presence of channel activity detection to improve the throughput of a single and multi-hop LoRaWAN network [22]. The performance of the proposed SF allocation algorithm shows improved throughput in comparison with SX1272 and SX1301 chips.

EXtending the performance of LoRa (EXPLoRa) [23] aims to improve the data extraction rate by proposing two SF allocation approaches; EXploRa-SF and EXplora air-time (EXplora-AT). EXploRa-SF allocates SFs to the entire network by considering the best RSSI while EXplora-AT aims to allocate a fair SF based on the air-time to EDs in the network by considering an “ordered water-filling [24]” approach to guarantee low ToA and channel fairness. The performance of both methods has been compared to the adaptive data rate (ADR) scheme, where the results of EXploRa-SF show improved results compared to ADR. In contrast, EXplora-AT shows even better performance under a high traffic load. The performance of EXplora-AT has been further enhanced by EXplora K-means (EXplora-KM) and EXplora-Time symbol (EXplora-TS), as presented in [25]. EXplora-KM identifies the crowded regions having high collision probability, thus increasing SF. On the other hand, EXplora-TS considers the heterogeneous traffic load, where EDs having a higher payload sizer are served based on priority. The performance of these two approaches has been compared to EXplora-AT, where results show a 21% increase. The authors in [26] further improved the performance of EXplora-AT and EXploRa-SF by considering the capture effect. EXplora-Capture (EXplora-C) in [26] equalizes the ToA of UL packets, keeping the balance regarding SF allocation in a single and multi-GW environment, and takes the capture effect into account. The performance of the EXplora-C approach has been compared to EXplora-AT and EXploRa-SF, where EXplora-C shows improved results in terms of the data extraction rate. In particular, EXplora-C shows enhanced results of up to 38% on average over the legacy ADR mechanism.

To improve the power efficiency of ADR, a proposed ADR is presented in [27]. Firstly, it takes the average of the SNRs of *M* UL packets (i.e., *M=20*). Secondly, it takes the standard deviation of the SNRs of *M* UL packets. The performance of the proposed ADR shows better performance than ADR in terms of power efficiency. However, the proposed work in [27] completely ignores the slow convergence time of the ADR, which is affected by variable channel conditions [28,29,30]. Due to variable channel conditions, the ADR is not suitable when the EDs are mobile; therefore, the authors propose an enhanced ADR (E-ADR) in [31]. The E-ADR is primarily based on the trilateration technique, which aims to estimate the next position of a mobile ED with a predefined trajectory. In different use cases, the E-ADR reduces energy consumption and ToA due to quick adaption. However, E-ADR is solely based on a preset path of the EDs.

### 2.3. Mathematical Model-Based Approaches

Some mathematical models for LoRaWAN have been developed in the literature [32,33,34,35,36]. The authors in [32] aimed to analyze and evaluate the LoRaWAN channel access operation in terms of the PER and packet loss ratio (PLR) in a confirmed mode. The results in [32] reveal that PER and PLR increases with an increasing load in the network. The authors in [33] further enhanced the work presented in [32] by proposing a mathematical model based on retransmissions. The proposed model accurately estimates the PER based on the offered traffic. The proposed method was verified through simulation by finding the probabilities of the distribution of data rates. The simulation results show that by considering the retransmissions, the model accuracy is significantly improved. Furthermore, the model presented in [33] was enhanced in [34] by taking into account the capture effect along with retransmissions. The authors assumed that the different SFs are entirely orthogonal. The performance of [34] has been further enhanced by [35] by proposing MCS allocation to satisfy the different Quality of Service requirements of IoT applications. However, the reception paths at the GW and duty cycle limitations both at EDs and GW were neglected. To take into the reception paths and duty cycle limitations, the authors proposed a mathematical model in [36]. The performance evaluation of the proposed model shows an improved packet success ratio when compared with [34].

## 3. Link-Level Model

### 3.1. Assumptions and Performance Metrics

In this study, we consider class *A* EDs for the LoRaWAN, where N number of EDs are uniformly distributed around a single GW. An ED always initiates a transmission in the UL by randomly choosing a channel frequency from Ć, where $\text{Ć}\in \left\{868.1,\;868.3,\;868.5\right\}$ MHz. However, in the DL, the same SF used in the UL is assigned for RW1 and SF 12, and the 869.525 MHz channel is used for RW2 [37].

We consider the packet success ratio, average end-to-end, and average acknowledgment delays as LoRaWAN performance metrics in this study.

The packet success ratio (SR) is the ratio of the number of packets successfully received to the number of packets transmitted by the EDs. It is defined as:

(1) $$SR=\frac{N_{r}}{N_{s}},$$

where $N_{r}$ is the number of packets successfully received at the GW, and $N_{s}$ is the number of total sent MAC packets.

When an ED transmits a confirmed UL packet toward the NS, it expects to receive a DL ACK in one of the RWs. In the absence of the DL ACK, an ED re-transmits the same confirmed UL packet at least ACK_TIMEOUT seconds after the RW2, as presented in [1]. After retransmission, if ED receives the DL ACK from NS during its RW1, as soon as ED demodulates the DL ACK, it is free to transmit a new confirmed UL packet on a random channel. In a confirmed mode, if an ED receives a DL ACK from the GW, a packet is considered delivered. The average end-to-end delay $\left(\hat{\mu }D\right)$ and average ACK delay $\left(\hat{\mu }D_{ack}\right)$ of the confirmed UL packet can be computed using Equations (2) and (3):

(2) $$\hat{\mu }D=\frac{\sum \left(T_{r}-T_{s}\right)}{N_{r}},$$

where $T_{r}$ is the time a packet has completely arrived and successfully received at the GW, and $T_{s}$ is the time a packet leaves the ED queue for transmission:

(3) $$\hat{\mu }D_{ack}=\frac{\sum \left(T_{ack}-T_{tx}\right)}{N_{r}}\;,$$

where $T_{ack}$ is the ACK reception time measured at the ED and $T_{tx}$ is the time a confirmed UL packet was transmitted from the ED for the first time. The $\hat{\mu }D_{ack}$ is the delay time between the first transmission of a packet at the MAC layer and the moment the ED receives the corresponding ACK. It is averaged over the total number of MAC packets received during the duration of the experiment.

Besides, in the unconfirmed mode, if a GW correctly receives a packet, then it is determined to be delivered. On the other hand, in the confirmed mode, a packet is considered delivered if the corresponding ED receives a DL ACK confirmation from the GW. Both $\hat{\mu }D$ and $\hat{\mu }D_{ack}$ are limited to the confirmed mode only. Moreover, the symbols used in the link-level model with descriptions are highlighted in Table 1.

### 3.2. Link Measurement Model

The link measurement model is primarily based on [5]. It considers the impact of the propagation on the signal strength, considering small-scale fading and its influences. The received power ($P_{rx}$) at the GW can be computed by:

(4) $$P_{rx}=\frac{P_{tx}\times G_{ED}\times G_{GW}}{PL}{ℯ}^{\xi },$$

where $G_{ED}$ and $G_{Gw}$, respectively, represent the ED and GW antenna gains; $P_{tx}$ is the transmit power, and PL is the path loss. Equation (4) can be re-written in the logarithmic domain, with $10\xi \log_{10}ℯ=4.34\xi$, we get:

(5) $$P_{rx}^{dB}=P_{tx}^{\mathrm{dB}}+G_{ED}^{\mathrm{dB}}+G_{GW}^{\mathrm{dB}}-PL^{dB}+4.34\xi ,$$

where $PL^{dB}$ is obtained by combining both the propagation and building penetration losses. The ${ℯ}^{\xi }$ in Equation (4) is the log-normal shadowing (or log-distance path loss) component, i.e., 4.34ξ ~ ℕ(μ, σ^2^), where μ = 0 is the mean captured in the PL, and σ represents the depth of shadowing, which is the logarithmic standard deviation (4<σ <12). During the simulation experiment, we consider the value of σ=6 dB, as presented in [2]. It is well known that the RSSI varies because of the objects obstructing the propagation path between an ED and a GW. In this paper, we consider both log-distance (*LOG*) and Okumura–Hata (*OH*) models, as presented in [21,22]. Additionally, we add building penetration loss caused by the signal attenuation due to the external wall, internal wall, and floors of the building [23,24].

#### Correlated Shadowing

The correlated shadowing generation model is mainly based on the decaying exponent of distance (i.e., distance-only model), as described in [38]:

(6) $$P_{rx\left(i,j\right)}\left(d_{i,j}\right)=e^{-\frac{d_{i,j}}{d_{0}}},$$

where $P_{rx\left(i,j\right)}$ is the measured received power at $ED_{j}$, when a packet is transmitted by $ED_{i}$; $d_{i,j}\;$ is the distance between two EDs (i  and j); and $d_{0}$ is a de-correlation distance parameter that is tunable and always greater than zero. The $d_{0}$ was set to 110 m in [2,39]. The shadowing values of EDs, which are not closely positioned on a vertex of the grid, are interpolated using an exponential covariance function described in [40]. In addition, the correlated shadowing in wireless communication is generally classified into two cases, and its implementation details are described in [5]:

1. When an ED transmits a packet to a GW, it is anticipated that the shadowing experienced by the GW is correlated with another shadowing disturbance at any other ED, which is “near” to it. This phenomenon is dependent on $d_{i,j}$ and has been demonstrated with an exponential function in [41].
2. If two EDs near each other transmit, it is expected that the shadowing values are correlated at the GW. This consequence is the site-to-site cross-correlation, as described in [42].

The model shown in Equation (6) captures the first case accurately and indicates that a GW “observes” two correlated shadowings caused by adjacent EDs. This observed shadowing caused by nearby EDs is used in the grid for every similar point. It results from the fact that signals of two adjacent EDs are mutually affected. In addition, the amount of shadowing that the GW experiences will be the same for the signals of two nearby EDs; therefore, the signal losses are correlated [43].

### 3.3. Link Performance Model

The link performance model targets abstraction of the real operation of the physical layer. More precisely, the model summarizes the performance of the Semtech SX1301 [44], SX1272 [45] chips commonly used in GW and EDs, respectively. The link performance model emulates their parallel decoding, sensitivity, and interference resistance performances.

#### 3.3.1. Receiver Sensitivity

Table 2 shows the SF sensitivities of GW ($S_{g}$) and ED ($S_{e}$) [44,45,46], respectively. For both the EDs and GW, the sensitivity decreases with an increasing SF. A packet can be detected by a device if its $P_{rx}$ is above the sensitivity level. In our experiment, we assume that the $P_{rx}$ of a packet is constant during the whole experimental period. If the power is sufficient to start the decoding process, a packet is assumed to be decodable till the end of the reception. Lastly, it is assumed that if two weak signals from the EDs arrive concurrently at the GW, they cannot be perceived as decodable even if the sum of their powers is above the sensitivity; this is owing to the fact that the signals interfere [5].

In our experiment, destructive interference is assumed, and the packet would be lost even if the receiver is locked onto it. The receiver sensitivity is dependent on the choice of SF (i.e., 7 to 12), as given by [44]:

(7) $$\mathrm{Sensitivity}\;\left(\mathrm{dBm}\right)=-174+10\log_{10}\left(BW\right)+NF+SNR.$$

where, in Equation (7), –174 dBm is the thermal noise computed for 1 Hz of BW; NF represents the noise margin at GW (6 dB) [44]; and SNR is the signal to noise ratio for a given SF, as shown in Table 3 [45].

#### 3.3.2. Interference Model

The LoRaWAN uses ALOHA as the channel access mechanism, where EDs do not listen to the channel before transmitting a packet. This mechanism causes a collision if multiple EDs transmit at the same time over the same channel. Therefore, the LoRaWAN network suffers from two types of collisions owing to multiple SFs, which are as follows: (1) Two or more packets with the same SF collide with each other over the same channel, and (2) packets with different SFs collide over the same channel [4]. These SF interferences cause a low SINR. By using the interference model described in [5], the SINR anticipated by the GW of Ć ($\gamma_{\text{Ć}}$), as given by:

(8) $$\;\gamma_{\text{Ć}}=\frac{P_{ℽ},r}{\sigma^{2}+{\mathbb{P}}_{Ꞷ}},$$

where $P_{ℽ},r$ is the power of a packet under observation and ${\mathbb{P}}_{Ꞷ}$ is the cumulative power, as presented in [5].

**Figure 1:**
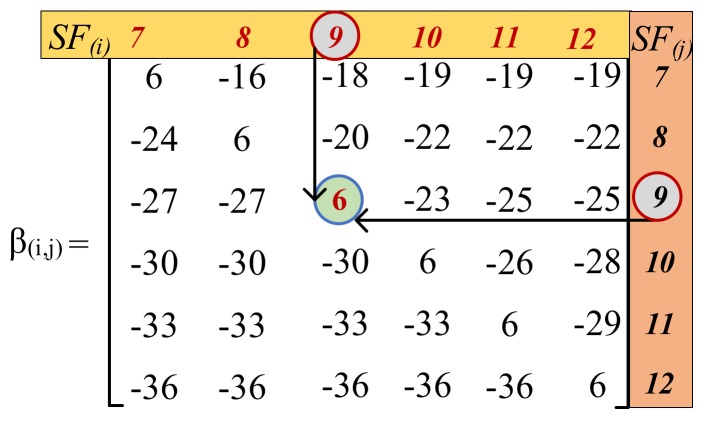
SINR threshold for different spreading factor combinations.

When two packets are received at a GW using $SF_{\left(i,j\right)}$ (i.e., $SF_{\left(i\right)}=9\;and\;SF_{\left(j\right)}=9$, as shown in Figure 1), it is considered successful if $\gamma_{\text{Ć}}\geq \beta_{\left(i,j\right)}$, where $\beta_{\left(i,j\right)}\;$(in dB unit) is a threshold corresponding to an acceptable SINR [47].

The SINR margin elements contained in $\beta_{\left(i,j\right)}$ (in dB) are calculated, assuming that the two packets are completely overlapped. However, in general, packets are not entirely overlapped. For this reason, the interfering power must be equalized in order to compute the $\gamma_{\text{Ć}}$ value [5,48]. It is assumed that, in general, the interfering energy of the mutual signal and the interferer can “spread out” on the useful signal as computed using Equation (8). If the interference is concentrated on a few consecutive symbols, assuming that a good interleaver will spread it out and allow the error correction code (i.e., the coding rate employed by LoRa CSS modulation, 4/5 in our case) to eventually correct the error caused by the interferer [49].

Based on the elements in $\beta_{\left(i,j\right)}$, we observed that one of two signals employing the same SF with the corresponding received power could be correctly received if both signals overlap for a small amount of time and their respective $\gamma_{\text{Ć}}$ after equalization is greater than 6 dB. The same observation is realized in [50]. The term “equalization” refers to the process of multiplying SNR of an interferer signal with the overlapping time, divided by the duration of the desired signal [48].

## 4. Proposed Spreading Factor Allocation Schemes

### 4.1. Channel-Adaptive SF Recovery Algorithm at the ED Side 

**Figure 2:**
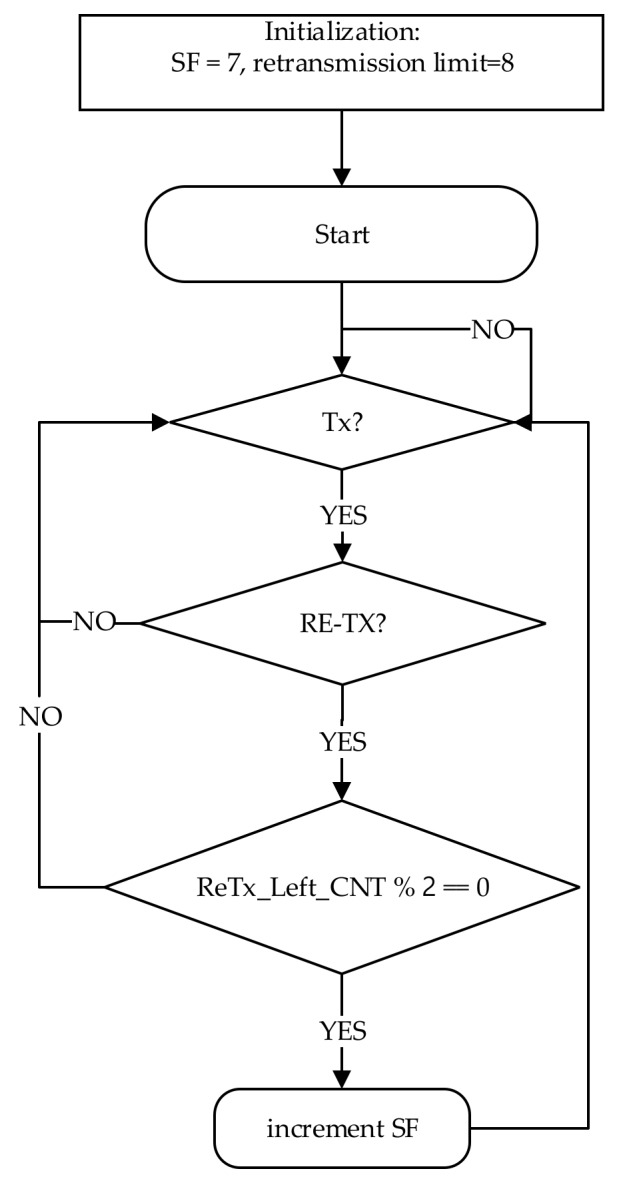
Typical SF scheme at the ED side [1,2,3].

According to the LoRaWAN specification [1], if an ED has not received an ACK in both RWs, it must wait for at least ACK_TIMEOUT  seconds before starting retransmission. Based on retransmissions, a typical SF management scheme suggests incrementing by one SF, each time two successive retransmissions fail and maintains that SF during the following transmissions of the ED, as shown in Figure 2 [1,2,3]. As mentioned, the higher SF increases the ToA, and thus the network suffers from high $\hat{\mu }D$ and $\hat{\mu }D_{ack}$ delays. Because the transmission failure may happen due to collisions, we could use a smaller SF to enhance the $P_{SR}$, as described in [1,2]. If the following conditions are satisfied, the SF is incremented by one, as shown in Figure 2.

1. The SF is smaller than the Max_SF.
2. The ED has not already performed the maximum number of retransmissions.
3. The number of ReTx_Left_CNT is a multiple of two.

In a massive LoRaWAN network, only incrementing the SF can create a situation where most of the EDs switch their SF to higher values, resulting in a high ToA and hence, a collision possibility [37]. Besides, the UL transmission becomes a bottleneck for dense LoRaWAN deployments, wherein the packet loss rate increases with the high traffic load situation. As a result, the power depletion of each ED causes a reduction of their battery lifetimes. Hence, in such cases, EDs should decrease their corresponding SF to lower the ToA if they can still reach the GW.

**Figure 3:**
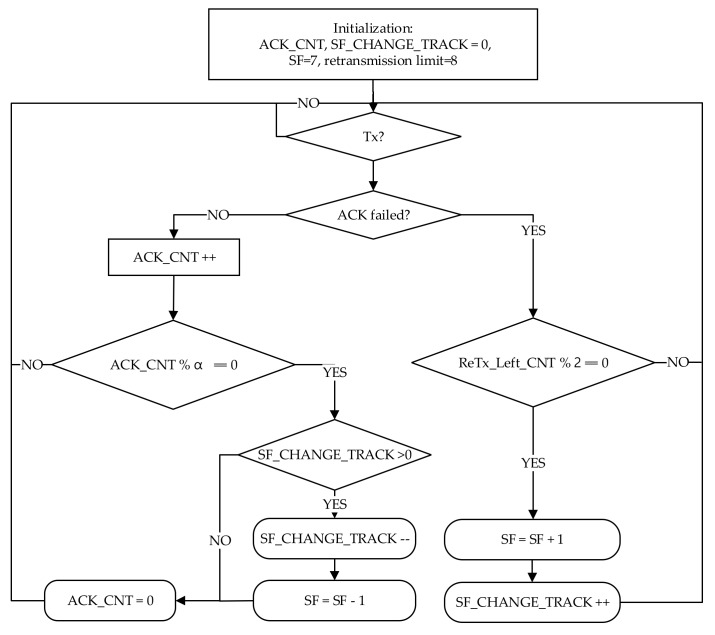
Proposed channel-adaptive SF scheme on the ED side.

The proposed channel-adaptive SF scheme is based on a typical SF management scheme [1,2,3]. The proposed channel-adaptive SF scheme is triggered after the retransmission is initiated from the ED. When the transmission starts, the proposed scheme checks for ACK failure. If the ACK failure is detected (i.e., no ACK is received in either of the RWs), then the next UL packet will be sent as a retransmission. If ReTx_Left_CNT is a multiple of two, the recovery SF scheme decides to increase the SF to a higher value, as in the typical SF assignment scheme. The SF is increased because of the ACK failure caused by packet congestion and unreliable wireless channel status. However, in the proposed channel-adaptive SF, as shown in Figure 3, if any changes are detected to the SF, a counter, denoted as SF_CHANGE_TRACK, keeps a count of the SF change. 

On the other hand, if transmission of a packet is successful (i.e., the ACK was successfully received), the algorithm keeps track of the successfully received ACKs by using a counter, ACK_CNT. If ACK_CNT reaches α, the network reliability is satisfied. Furthermore, the SF_CHANGE_TRACK value is verified if it is greater than zero. It shows that SF is higher than seven (Min_SF<SF≤ Max_SF). Therefore, it can be decremented, and the proposed channel-adaptive SF scheme lowers the SF further to decrease the ToA when the channel is determined to be stable. The detailed working of the recovery SF scheme and symbols are shown in Figure 3 and Table 4, respectively.

The proposed scheme can be further enhanced by taking into account the two types of DL PER: (1) $PER_{DL}^{UL}$, which is the number of correctly received DL ACK packets to the number of confirmed UL packets; and (2) $PER_{DL}^{ACK}$, which is the ratio of successfully received DL ACK packets to the number of sent DL ACK packets. These PER methods can be used as a decision metric to decrement the SF instead of ACK_CNT on the left side of the proposed scheme, as shown in Figure 3.

### 4.2. Proposed Distance-Based SF Assignment Algorithm (ED Sensitivity) 

During the initial deployment, each ED is assigned with an SF based on the ED sensitivity, as shown in Algorithm 1. In the proposed method, first, we find the distance between ED and GW using a similar approach (i.e., Euclidean distance), as presented in [7]. Secondly, we measure $P_{rx}$ at GW (i.e., a GW would receive from ED), assuming a time-independent and symmetric link, where the channel uses the same path loss model for both UL and DL transmissions [5]. When the value of $P_{rx}$ is measured, it is checked against $S_{e}$, as specified in Table 2. If the condition holds, each ED picks an SF based on $S_{e}$ using getSF function, to minimize the ToA and lower the probability of packet collisions. Furthermore, in a realistic environment, shadowing conditions change with time. However, in this work, the channel is time-independent, thus when SFs are assigned, no further adaptation is required.
**Algorithm 1.** SF assignment based on ED sensitivity.
**INPUT: **$S_{e}=$ ED sensitivities from**OUTPUT:** Assignment of spreading factors to N number of EDs**for** i in NCalculate $P_{rx}$ (i.e., a GW would receive from ED)$P_{rx}=getRecvPower\left(ED_{i}\right)$ **if** ($P_{rx}$ ˃ $S_{e}$)  $ED_{i}\;\leftarrow getSF\left(P_{rx}\right)$ **end****end**

## 5. Experimental Results and Analysis

The performance analysis of the LoRaWAN under a realistic channel model and high-density urban area topology is presented in this section in terms of SR, $\hat{\mu }D$, and $\hat{\mu }D_{ack}$ delays. The simulations were carried out in the NS-3 network simulator, where N number of EDs are uniformly distributed in a single GW environment. During the simulation, every ED transmits a packet of 51-byte length with a $P_{tx}$ of 14 dBm for 24 h of simulation time at a randomly chosen channel from Ć for the UL transmission. Each simulation is run 10 times with different seeds, and the average results are shown. The remaining simulation parameters are shown in Table 5. The rest of this section presents the analysis of the effects of interference occurring at different conditions of SFs, SR, $\hat{\mu }D$, and $\hat{\mu }D_{ack}$ delays.

### 5.1. Intra- and Inter-SF Interference

**Figure 4:**
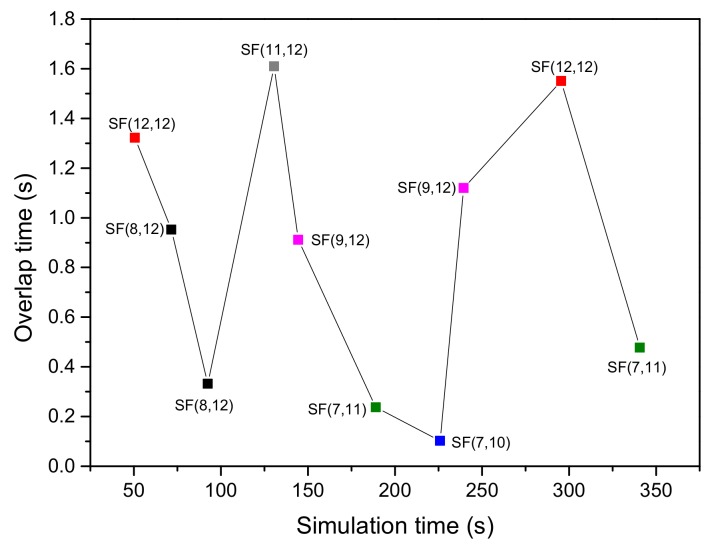
Collision overlap time of intra- and inter-SF interference using $SF_{\left(i,j\right)}$.

The analysis of intra- and inter-SF interferences during 600 s of simulation time is shown in Figure 4. The simulation scenario includes 100 EDs in a radius of 5 km, where SFs are assigned based on our proposed ED sensitivity-based approach. Every ED transmits a single packet of 51 bytes for the UL in unconfirmed mode using the 125-kHz bandwidth. The analysis in Figure 4 depicts collision overlap time occurred due to the collision of packets with the same and different $SF_{\left(i,j\right)}$ over the same channel. Considering the interference among the same SF, at the simulation time of 50 s, two packets using $SF_{\left(i,j\right)}=\;\mathrm{SF}\;12\;$(SF 12 with the ToA equals to 2.46 s) arrive at the GW with an overlap of 1.3 s. After computing $\gamma_{\text{Ć}}$ (which is –5.39 dB), the respective SINR after equalization is not greater than 6 dB, resulting in a packet loss [11,50]. Let us consider the case where a collision occurs between two packets with different SF (i.e., $SF_{\left(i\right)}=9$ and $SF_{\left(j\right)}=$ 12). This collision occurs at the simulation time of 240 s and lasts up to 1.1 s. The $\gamma_{\text{Ć}}$ value for this collision is –86.63 dB, which is smaller than $\beta_{\left(i,j\right)}$ (i.e., –36 dB), resulting in a packet loss. From Figure 4, we can observe that both the intra- and inter-SF collisions are certainly an issue in LoRaWAN networks. Besides, the recent works also confirmed the fact that SFs are not completely orthogonal among themselves [10,11,12,13,14]. We also observed that if two packets using the same SF overlap on the same channel for a small amount of time and their respective SINR is greater than $\beta_{\left(i,j\right)}$, then both packets can be correctly decoded.

### 5.2. Proposed Channel-Adaptive SF Scheme in a Confirmed Mode

**Figure 5:**
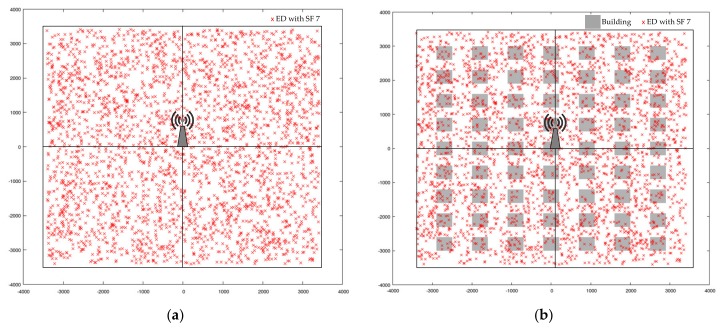
ED distribution under the condition of GW radius = 3410 m: (**a**) *LOG* path loss only; (**b**) *LOG*, shadowing, and building penetration losses.

The proposed scheme was evaluated under a *LOG* path loss and an urban environment, as shown in Figure 5a,b, respectively. As shown in Figure 5b, a square-shaped building based on the Manhattan layout model and correlated shadowing model was adapted in this work [51]. If an ED is randomly deployed inside this building, the transmission from this ED will suffer from high penetration losses. The building parameters utilized in an urban environment are presented in our previous work [52]. When the channel has fading, shadowing, and attenuation caused by the urban environment, it is more difficult for the EDs to gain connectivity with a GW, thereby resulting in many EDs going out of range [53]. For this reason, the GW radius is limited to 3410 m. During the simulation experiment, ED transmits a single packet in UL every 1 h, including a random time distributed between 0 and 1000 s to avoid simultaneous packet transmission [54].

**Figure 6:**
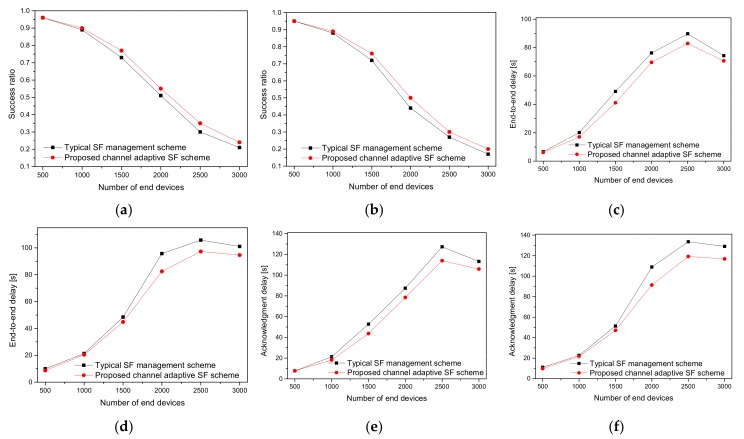
Performance of the SF assignment schemes under the various conditions of channel and traffic loads: (**a**) SR under only the path loss; (**b**) SR under the path loss, shadowing, and other RF features; (**c**) end-to-end delay under only the path loss; (**d**) end-to-end delay under the path loss, shadowing, and other RF features; (**e**) ACK delay under only the path loss; (**f**) ACK delay under path loss, shadowing, and other RF features.

Figure 6 presents the performance analysis of the typical and proposed channel-adaptive SF schemes. As shown in Figure 6a,b, the SR generally decreases with a massive number of EDs joining the network in both schemes. Therefore, when an ED does not receive an ACK, it transmits at low data rates after a few retransmissions. Owing to the higher ToA, the collision probability increases. When a packet collision happens, the two EDs involved in the collision schedule a retransmission time imposed by the duty cycle restriction. The higher the number of retransmissions, the more packets are lost because of interference [2]. That is, in a massive LoRaWAN network, a higher traffic load increases the chances of collision probability, and most of the RW is missed due to the duty cycle limitations [8,37]. As a result, more EDs with the SF management scheme transmit with high SFs and cause more congestion, and the network loses the advantage of orthogonality between different SFs. However, the proposed channel-adaptive SF scheme increases the data rate when ACK failures are reduced, thus lowering the SF and yielding a better SR of up to 7.5% and 7.8% for Figure 6a,b, respectively. Furthermore, the performance of both the SF approaches is lower in Figure 6b than in Figure 6a. This is due to the signal strength being significantly decreased by the building penetration losses, which results in a lower SR. In the case of an urban environment, many of the EDs cannot reach the GW due to the unfavorable channel that leads to a high packet loss. As these EDs stay active and cause interference to the nearby EDs, the scalability of the LoRaWAN is retained. Another possibility is that the duty cycle limitations imposed by the LoRaWAN do not allow such transmissions if the EDs reach the maximum allowed time. Therefore, the $\hat{\mu }D$ and $\hat{\mu }D_{ack}$ delays go up in both the cases as shown in Figure 6c,d, and Figure 6e,f, respectively.

### 5.3. ED Sensitivity-Based SF Allocation in a Confirmed Mode

**Figure 7:**
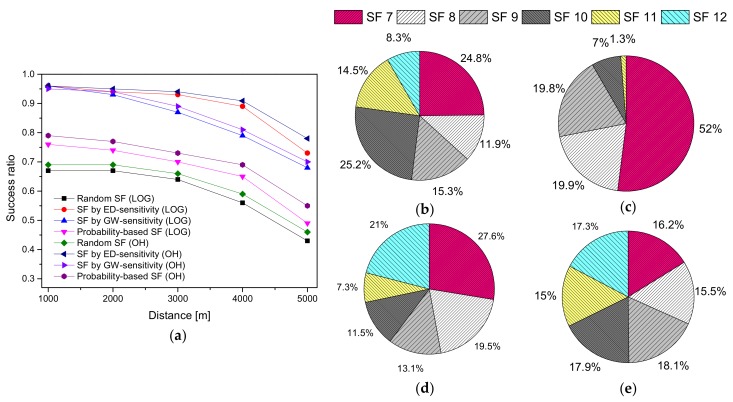
The success ratio and SF assignment ratio of the ED sensitivity, GW sensitivity, random-based, probability-based SF allocation schemes by varying the distance in an urban environment: (**a**) SR under LOG and OH; (**b**) SF assignment ratio of ED sensitivity; (**c**) SF assignment ratio of GW sensitivity; (**d**) SF assignment ratio of probability-based SF; (**e**) SF assignment ratio of random-based SF.

In Figure 7, the number of EDs is kept fixed at 1000, while varying the distance in an urban environment. Figure 7a shows a declining trend in SR, as the distance is increased. The decreasing trend of the SR is because of the high interference among the SFs or the unreachability of the GW [55]. In the case of the proposed ED sensitivity method, the distribution of SFs is very uniform, as shown in Figure 7b, resulting in an enhanced SR of up to 5% on an average as compared to GW sensitivity method. However, the performance of the ED sensitivity decreases gradually when the signal strength of an ED increases or decreases, as the distance is reduced or increased from the GW, respectively. This is because of the assignment of the SFs based on the sensitivities of the EDs, wherein the UL packets are successful, but the ACKs are lost. Therefore, EDs retransmit packets, causing significant congestion in the network and resulting in packet loss.

On the other hand, in Figure 7a, the GW sensitivity SF assignment scheme (uses sub-optimal fixed-width SF rings) shows a decreasing tendency in SR with increasing distance. More than 50% of the end devices use SF 7, as shown in Figure 7c, thus leading to substantial packet loss owing to the interference and retransmission. The probability-based SF method is primarily based on a theoretic distribution of data rates, as presented in [33]. The corresponding SF assignment ratio of the probability-based SF allocation method is shown in Figure 7d. The EDs are forced to use a given SF, resulting in a significant impact on the SR. Additionally, we found a similar observation regarding [33], as presented in [2]. Lastly, a random SF allocation algorithm performs worse than ED and the GW sensitivities methods in terms of SR because of the unfair distribution of SF to the EDs. Therefore, it is a sub-optimal choice for the SF assignment [56].

**Figure 8:**
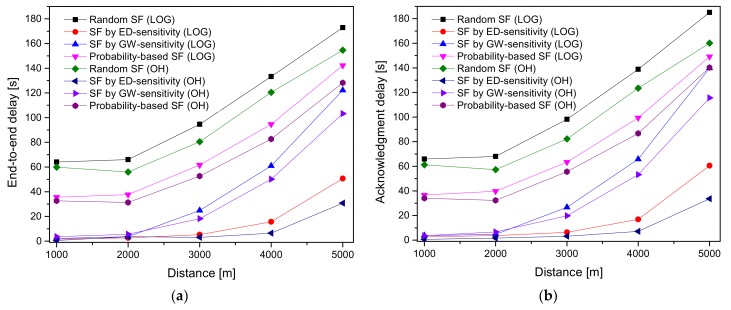
Delay analysis of the ED sensitivity, GW sensitivity, and random-based SF allocation schemes under the LOG and OH path loss models by varying the distance in an urban environment: (**a**) end-to-end delay (**b**) ACK delay.

Figure 8a,b present the $\hat{\mu }D$ and $\hat{\mu }D_{ack}$ delays in the confirmed mode with a maximum of eight transmissions. In general, $\hat{\mu }D$ and $\hat{\mu }D_{ack}$ delays increase by increasing the distance, as the SR declines, and more retransmissions are needed to deliver a packet successfully. $\hat{\mu }D$ and $\hat{\mu }D_{ack}$ delays are maximum at GW radius = 5000 m, which is because of the number of interfered packets increases, resulting in retransmissions. $\hat{\mu }D_{ack}$ correspondingly considers the time for the ACK reception; hence, the results show a higher $\hat{\mu }D_{ack}$ as compared to $\hat{\mu }D$.

### 5.4. ED Sensitivity-Based SF Allocation in an Unconfirmed Mode

**Figure 9:**
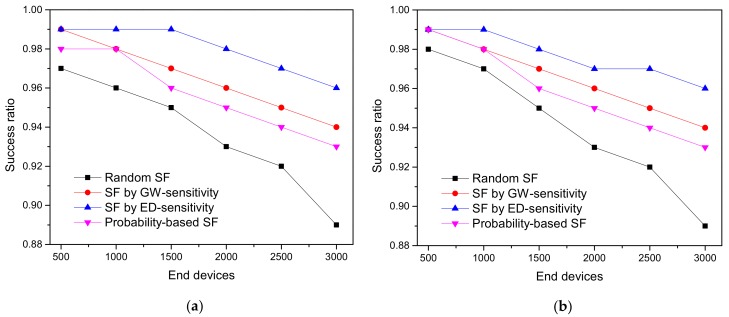
The success ratio of the SF allocation schemes in the unconfirmed mode under the condition of GW radius = 3410 m: (**a**) SR under LOG; (**b**) SR under OH.

The SR of ED sensitivity, GW sensitivity, and the random SF allocation-based schemes in an unconfirmed mode with log-normal shadowing and Okumura–Hata propagation models are presented in Figure 9. In comparison with the confirmed mode, the unconfirmed mode performs exceptionally well under a traffic load of 24 packets/day. In fact, with confirmed communication, the network traffic is increased by both the ACK messages sent in RWs and the retransmissions. This causes an avalanche effect, as a packet loss causes retransmissions that lead to an increase in the interference. Furthermore, in the unconfirmed mode, an unsuccessful message delivery could only be caused by a data packet loss.

## 6. Conclusions

In this study, we analyzed the intra- and inter-SF collisions and proposed two SF assignment schemes for the confirmed and unconfirmed modes in LoRaWAN. Through simulation results, we observed that the transmissions arriving at the GW with the same or different SFs could be correctly received, only when their respective SINR values are above a certain threshold. It was also shown that a higher SF is highly vulnerable to interference due to high ToA. Therefore, assigning higher SFs to EDs far away from the GW might not necessarily solve the network congestion as both the intra- and inter-SF interferences exist in the LoRaWAN networks. The first scheme tackled the higher SF issue by decreasing it in the worst case and certainly enhanced the SR, $\hat{\mu }D$, and $\hat{\mu }D_{ack}$ delays in comparison with the existing typical SF scheme. Furthermore, the second scheme played a vital role in assigning different SF to EDs in the same region; as a result, it reduced the impact of interference on the network and enhanced the success ratio. We believe that the two proposed SF assignment methods can be used for IoT application services, offering a high transmission success ratio without the sacrifice of energy consumption and computation cost.

## Figures and Tables

**Table 1:** Symbols utilized in the link-level model along with their description.

Symbols	Description
SR	packet success ratio
$N_{r}$	number of packets successfully received at the GW
$N_{s}$	number of total sent MAC packets
$\hat{\mu }D$	average end-to-end delay
$T_{r}$	the time a packet has completely arrived and successfully received at the GW
$T_{s}$	the time a packet leaves the ED queue for transmission
$\hat{\mu }D_{ack}$	average ACK delay
$T_{ack}$	ACK reception time measured at the ED
$T_{tx}$	the time a confirmed UL packet was transmitted from the ED for the first time
$P_{rx}$	the received power at the GW
$P_{tx}$	transmit power
$G_{ED}$	ED antenna gain
$G_{GW}$	GW antenna gain
${ℯ}^{\xi }$	log-normal shadowing (or log-distance path loss) component
PL	path loss
$P_{rx\left(i,j\right)}$	the measured received power at $ED_{j}$, when a packet is transmitted by $ED_{i}$
$d_{i,j}$	distance between two EDs (i and j)
BW	bandwidth
NF	noise margin at GW
Ć	number of default channels in the EU region
$\gamma_{\text{Ć}}$	SINR anticipated by the GW
$P_{ℽ},r$	power of a packet under observation
${\mathbb{P}}_{Ꞷ}$	cumulative power
$\beta_{\left(i,j\right)}$	threshold corresponding to an acceptable SINR

**Table 2:** Sensitivities (dBm) of end devices and gateway for 125 kHz bandwidth.

SF	BW [kHz]	$S_{g}$	$S_{e}$
12	125	–142.5	–137.0
11	125	–140.0	–135.0
10	125	–137.5	–133.0
9	125	–135.0	–130.0
8	125	–132.5	–127.0
7	125	–130.0	–124.0

**Table 3:** The LoRa demodulator signal to noise ratio for spreading factors.

SF	SNR (dB)
12	–20
11	–17.5
10	–15
9	–12.5
8	–10
7	–7.5

**Table 4:** Symbols used in the typical SF and proposed channel-adaptive SF schemes.

Symbols	Description	Value
ACK_TIMEOUT	waiting time before retransmission	1 to 3 s
ReTx_Left_CNT	number of retransmissions left to complete the packet transmission	multiple of 2
Max_Tx	maximum number of transmissions allowed	8
ACK_CNT	number of DL ACK packets received	32
SF_CHANGE_TRACK	number of times SF has been changed during the simulation time	6
Max_SF	maximum SF	12
Min_SF	minimum SF	7
α	ACK_CNT threshold value	32
$PER_{DL}^{UL}$	$\frac{\mathrm{DL}\;\mathrm{ACK}\;}{No.\;of\;UL\;packets\;transmitted}$	-
$PER_{DL}^{ACK}$	$\frac{\mathrm{DL}\;\mathrm{ACK}\;}{No.\;of\;DL\;ACK\;packets\;transmitted}$	-

The choice of ReTx_Left_CNT and Max_Tx is based on [2,3]. Whereas the choice of SF_CHANGE_TRACK and α is based on hit and trial method.

**Table 5:** Parameters utilized in simulation.

Parameters	Values
Simulation time [h]	24
Uplink interval	24 packets/day
GW radius [m]	3410
Number of GWs	1
Receive paths at GW	8
Packet size [bytes]	51
Mode of communication	confirmed and unconfirmed
Maximum packet transmission limit	8
GW antenna height [m]	15
ED antenna height [m]	1.2
Path loss exponent	3.76
Path loss model	log-distance & Okumura-Hata
Shadowing	$d_{0}$ = 110 m, variance = 6 dB [2,38]
Buildings	height = 6 m, number of floors = 2 [51]

## Nomenclature

LoRaWAN	long-range wide area network
SF	spreading factor
ED	end device
IoT	internet of things
MAC	media access control
CSS	chirp spread spectrum
DR	data rate
RW	receive window
DL	downlink
NS	network server
UL	uplink
ToA	time-on-air
SNR	signal-to-noise ratio
GW	gateway
RF	radio frequency
AWGN	additive white Gaussian noise
CIR	cumulative interference ratio
RS-LoRa	reliability and scalability of LoRa
RSSI	received signal strength indicator
ASFS	adaptive spreading factor selection
EXPLoRa	EXtending the performance of LoRa
EXplora-AT	EXplora air-time
EXplora-KM	EXplora K-means
EXplora-TS	EXplora-Time symbol
E-ADR	enhanced ADR
PLR	packet loss ratio
MCS	modulation and coding schemes
QoS	quality of service
OH	Okumura-Hata
LOG	log-distance
ADR	adaptive data rate
PER	packet error rate
